# Supplementary material for: Surveillance for Enteroviruses Associated with Hand, Foot, and Mouth Disease, and Other Mucocutaneous Symptoms in Spain, 2006–2020
Source: Viruses. 2021 Apr 28;13(5):781. doi: 10.3390/v13050781 (PMC8146579; doi:10.3390/v13050781)
Supplement: Supplementary file 1 [file viruses-13-00781-s001.zip › viruses-1152365-supplementary.pdf]

Table S1. CVA6 sequences included in the phylogenetic analysis and obtained from GenBank

Strain	Country	Date	GenBank accession no.
CV-A6-GDULA(RC)	USA	1949	AY421764
JQ364866/CHN/1992	CHN	1992	JQ364886
JQ364887/CHN/1996	CHN	1996	JQ364887
354/CA6/Shiga/1999	JPN	1999	AB114114
P-536/CA6/Kanagawa/2000	JPN	2000	AB114099
NOR02-319/2	NOR	2002	DQ317276
NOR03-1014	NOR	2003	DQ317282
KOR03 DK3-102	KOR	2003	AY748797
TW 2004-02892	TW	2004	EU908165
Fukuoka City2005-85	JPN	2005	AB234336
Fukuoka City2005-86	JPN	2005	AB234337
Fukuoka City2005-87	JPN	2005	AB234338
TW 2005-07594	TW	2005	EU908169
LC421554/JPN/2006	JPN	2006	LC421554
TW 2006-03257	TW	2006	EU908148
JN203517/IND	IND	2007	JN203517
GRC 48.07/2007	GRC	2007	FM878831
TW 2007-00720	TW	2007	EU908152
FR797988/ESP/2008	SP	2008	FR797988
LC421627/JPN/2008	JPN	2008	LC421627
FIN08/So2413	FIN	2008	GU248481
FIN08/We2417	FIN	2008	GU248480
FIN08/No2426	FIN	2008	GU248482
FIN08/We2419	FIN	2008	GU248483
FR797987/SP/2008	SP	2008	FR797987
ESP08/54594	SP	2008	FR796477
ESP08/1215	SP	2008	FR797986
ESP08/1023	SP	2008	FR797984
IND 0913543-09	IND	2009	HM190277
IND 099151-09	IND	2009	HM190275
HE572935/FRA/2010	FRA	2010	HE572935
JQ946053/TW/2010	TW	2010	JQ946053
MF596066/CHN/2010	CHN	2010	MF596066
CF152047 FRA10	FRA	2010	HE572913
CF194018 FRA10	FRA	2010	HE572937
TH/CU84/A/2010	THA	2010	KX212342
SHAPHC1253F/SH/CHN/2010	CHN	2010	JN316021
CF147014 FRA10	FRA	2010	HE572909
UK/V1/Ed/2010	UK	2010	KP129348
CHN/HK456845/NOV2010	CHN	2010	MH049854
JPN/2134/2010	JPN	2010	LC421636
JPN/2182/2010	JPN	2010	LC421638
TW/409/A/JUN2010	TW	2010	JQ946055
SHAPHC1283F/SH/CHN/2010	CHN	2010	JN316023
CF155023 FRA2010	FRA	2010	HE572915
SHAPHC1232T/SH/CHN/2010	CHN	2010	JN316017

TH/CU83/A/2010	THA	2010	KX212341
HE572938/FRA/2010	FRA	2010	HE572938
JX154929/CHN/2010	CHN	2010	JX154929
GU59482-OCT2010	SP	2010	KC688834
CA70062-NOV2010	SP	2010	KC688835
MA69993-NOV2010	SP	2010	KC688836
UK/V1/Ed/2011	UK	2011	KP144349
23-0215/osaka.JPN/2011	JPN	2011	AB688679
shizuoka-19/2011	JPN	2011	AB649291
shizuoka-9/2011	JPN	2011	AB649287
shizuoka-1/2011	JPN	2011	AB649286
23-0214/osaka.JPN/2011	JPN	2011	AB688677
TH/CU137/OCT2011	THA	2011	JX626314
TW/1537/A/2011	TW	2011	JN582001
SHAPHC3411/SH/CHN/11	CHN	2011	AFV26058
SHAPHC3468/SH/CHN/11	CHN	2011	JX495149
GU30838-JUN2011	SP	2011	KC688837
VI46737-SEP2011	SP	2011	KC688838
VI46774-SEP2011	SP	2011	KC688839
VI46750-SEP2011	SP	2011	KC688840
PA46098-SEP2011	SP	2011	KC688841
VI55717-OCT2011	SP	2011	KC688842
MA54125-NOV2011	SP	2011	KC688843
MA54129-NOV2011	SP	2011	KC688844
VI55713-NOV2011	SP	2011	KC688845
MA56722-NOV2011	SP	2011	KC688846
TH/CU/179/JUN2012	THA	2012	JX556432
TH/MUMT01/OCT2012	THA	2012	KX372340
CHN/HK418670/APR2012	CHN	2012	MH049857
CHN/SZ/JB141210046/JUN2012	CHN	2012	MH321902
UK/V1/Ed/2012	UK	2012	KP144350
UK/V2/Ed/2012	UK	2012	KP129347
CHN/SH/4368/2012	CHN	2012	KJ541169
KX212514/THA/2012	THA	2012	KX212514
KC207848/CHN/2012	CHN	2012	KC207848
MA14329-MAR2012	SP	2012	KC688847
MA14335-MAR2012	SP	2012	KC688848
GC25060-MAY2012	SP	2012	KC688849
BA44008-MAY2012	SP	2012	KC688850
V23992-JUN2012	SP	2012	KC688851
MA29275-JUL2012	SP	2012	KC688852
V38215-OCT2012	SP	2012	KC688853
CA46827-NOV2012	SP	2012	KC688855
BA44190-NOV2012	SP	2012	KC688856
GU00153-DIC2012	SP	2012	KC688857
UK/V7/Ed/2013	UK	2013	KP144342
CHN/P115/2013	CHN	2013	KP289367
CHN/SZc173/2013	CHN	2013	KF682362
LC364117/JPN/2013	JPN	2013	LC364117
KJ865454/CHN/2013	CHN	2013	KJ865454

KY424374/CHN/2014	CHN	2014	KY424374
UK/V1/Gla/2014	UK	2014	KP144343
DE/G4/A/2014	GER	2014	KX212508
TH/CU1499/A/2014	THA	2014	KX212503
SE/116246/2014	SWE	2014	KX212488
TH/CU1555/A/2014	THA	2014	KX212404
DK/M22061/A/2014	DEN	2014	KX212411
DK/H36898/A/2014	DEN	2014	KX212416
CHN/M27-YN/2014	CHN	2014	LC412058
CHN/R109/YN/2014	CHN	2014	KY211739
KX212527/CHN/2014	CHN	2014	KX212527
DE/47066/2015	GER	2015	MH472799
KX212502/THA/2015	THA	2015	KX212502
UK/SCO-SW/2015	UK	2015	MH361015
TH/CU1624/A/2015	THA	2015	KX212394
TH/CU1665/A/2015	THA	2015	KX212388
JPN/FS212/Fukuoka/2015	JPN	2015	LC364161
CHN/LW03R/Shandong/2015	CHN	2015	KY126091
CHN/WH15066/Shandong/2015	CHN	2015	KY126092
LC421572/JPN/2015	JPN	2015	LC421572
MH539784/IND/2015	IND	2015	MH539784
KU708609/CHN/2015	CHN	2015	KU708609
JPN/2091/2016	JPN	2016	LC421576
DE/47914/2016	GER	2016	MH472790
CHN/SDTA/282/2016	CHN	2016	MH086214
CHN/R94-YN/2016	CHN	2016	LC412976
JPN/FS601/Fukuoka/2016	JPN	2016	LC364168
MH539786/IND/2016	IND	2016	MH539786
MF596136/CHN/2016	CHN	2016	MF596136
IT/DG04/2017	ITA	2017	MH371303
JPN/FS365/Fukuoka/2017	JPN	2017	LC364193
DE/43421/2017	GER	2017	MH472798
JPN/FS82/Fukuoka/2017	JPN	2017	LC364172
CHN/147/2017	CHN	2017	MG385798
CHN/91/2017	CHN	2017	MG385831
CHN/JN414/2018	CHN	2018	MK357085
CHN/A282-KM/2018	CHN	2018	LC516767
CHN/S3083/2019	CHN	2019	MN329130
CHN/B16/Shenyang/2019	CHN	2019	MK307022
